# Post-Irradiation Thymic Regeneration in B6C3F1 Mice Is Age Dependent and Modulated by Activation of the PI3K-AKT-mTOR Pathway

**DOI:** 10.3390/biology11030449

**Published:** 2022-03-16

**Authors:** Masaaki Sunaoshi, Benjamin J. Blyth, Yi Shang, Chizuru Tsuruoka, Takamitsu Morioka, Mayumi Shinagawa, Mari Ogawa, Yoshiya Shimada, Akira Tachibana, Daisuke Iizuka, Shizuko Kakinuma

**Affiliations:** 1 Department of Radiation Effects Research, National Institute of Radiological Sciences, Quantum Life and Medical Science Directorate, National Institutes for Quantum Science and Technology, 4-9-1 Anagawa, Chiba 263-8555, Japan; sunaoshi.masaaki@qst.go.jp (M.S.); benjamin.blyth@petermac.org (B.J.B.); shang.yi@qst.go.jp (Y.S.); tsuruoka.chizuru@qst.go.jp (C.T.); morioka.takamitsu@qst.go.jp (T.M.); shinagawa.mayumi@qst.go.jp (M.S.); ogawa.mari@qst.go.jp (M.O.); shimada.yoshiya@ies.or.jp (Y.S.); kakinuma.shizuko@qst.go.jp (S.K.); 2 Graduate School of Science and Engineering, Ibaraki University, 2-1-1 Bunkyo, Mito 310-8512, Japan; akira.tachibana.sci@vc.ibaraki.ac.jp

**Keywords:** age dependency, thymic regeneration, PI3K-AKT-mTOR signaling pathway, cell proliferation/survival, radiation-induced thymic lymphoma

## Abstract

**Simple Summary:**

Because children have a long life expectancy relative to adults and their tissues and organs are growing and developing rapidly, the risk of radiation carcinogenesis for children is considered higher than that for adults. However, the underlying mechanism(s) is unclear. To uncover the mechanism, we previously revealed that principal causative genes in mouse thymic lymphomas arising in irradiated infants or adults as *Pten* or *Ikzf1*, respectively, suggesting that cells with mutation in these genes might be the origin of lymphomas arising after irradiation depending on age at exposure. Here, we clarified the age-dependent differences in thymus-cell dynamics in mice during the initial post-irradiation period. Our results demonstrate that the dynamics of thymocytes during the post-irradiation period depends on the age at exposure. For irradiated infants in particular, the number of proliferating cells increase dramatically, and this correlate with activation of the PI3K-AKT-mTOR pathway. Thus, we conclude that the PI3K-AKT-mTOR pathway in infants contributed, at least in part, to thymus-cell dynamics through the modification of cell proliferation and survival after irradiation, which may be associated with the risk of *Pten* mutation-associated thymic lymphoma.

**Abstract:**

The risk of radiation-induced carcinogenesis depends on age at exposure. We previously reported principal causative genes in lymphomas arising after infant or adult exposure to 4-fractionated irradiation as *Pten* or *Ikzf1*, respectively, suggesting that cells with mutation in these genes might be the origin of lymphomas arising after irradiation depending on age at exposure. Here, we clarified the age-dependent differences in thymus-cell dynamics in mice during the initial post-irradiation period. The thymocyte number initially decreased, followed by two regeneration phases. During the first regeneration, the proportion of phosphorylated-AKT-positive (p-AKT^+^) cells in cell-cycle phases S+G2/M of immature CD4^−^CD8^−^ and CD4^+^CD8^+^ thymocytes and in phases G0/G1 of mature CD4^+^CD8^−^ and CD4^−^CD8^+^ thymocytes was significantly greater in irradiated infants than in irradiated adults. During the second regeneration, the proportion of p-AKT^+^ thymocytes in phases G0/G1 increased in each of the three populations other than CD4^−^CD8^−^ thymocytes more so than during the first regeneration. Finally, PI3K-AKT-mTOR signaling in infants contributed, at least in part, to biphasic thymic regeneration through the modification of cell proliferation and survival after irradiation, which may be associated with the risk of *Pten* mutation-associated thymic lymphoma.

## 1. Introduction

Because children have a long life expectancy relative to adults and their tissues and organs are growing and developing rapidly, the risk of radiation carcinogenesis for children is considered higher than that for adults [1]. An increased risk of leukemia—in particular acute lymphoblastic leukemia—in children has been reported in studies of atomic bomb survivors from Hiroshima and Nagasaki [2,3], suggesting the existence of an age involving an exposure-dependent mechanism for lymphomagenesis after radiation exposure. Our previous studies showed that, compared with adult mice, intestinal cells in infants are more resistant to radiation-induced apoptosis [4] and that the rate of radiation-induced hepatocyte proliferation is higher in irradiated infant mice than in irradiated adults [5]. Moreover, radioprotective cytokines such as *Csf2*, *Fgf1* and *Il1b* are secreted in adult bone marrow in response to irradiation, but this does not occur in infant mice [6]. These results suggest that age-dependent differences during the initial response to radiation exposure may affect carcinogenic susceptibility and its underlying mechanism. However, in radiation-induced mouse thymic lymphoma, which is considered a mouse model of human acute lymphoblastic leukemia, the dependence of carcinogenesis-related cell dynamics (i.e., changes in thymocyte number owing to cell death, differentiation, or proliferation) on age at exposure has not been clarified.

Mouse models of radiation-induced thymic lymphoma have long been used to elucidate the mechanisms of radiation carcinogenesis because thymic lymphoma occurs at high frequency in mice exposed to radiation weekly for four consecutive weeks starting at age 4–5 weeks [7]. To determine the molecular mechanism underlying radiation carcinogenesis, we previously reported the existence of mutations and genetic alterations in tumor-suppressor genes in mouse thymic lymphomas [8,9,10,11]. The incidence of radiation-induced lymphoma is higher when mice are irradiated at a young age (e.g., 5 weeks old) compared with those irradiated at an older age (18 months old) [12]. We previously studied mutations and genetic alterations in lymphomas arising after weekly irradiation of female B6C3F1 mice with 1.2 Gy of X-rays for four consecutive weeks starting during infancy (1 week old) or young adulthood (8 weeks old), revealing that the genes that were mutated in the lymphomas differed depending on age at exposure—even if lymphoma was similar in the two groups [13]. In particular, the tumor-suppressor gene *Pten* was frequently mutated in lymphomas induced by irradiation of infants, whereas the tumor-suppressor gene *Ikzf1* was frequently mutated in lymphomas arising after irradiation of adults. IKAROS, which is encoded by *Ikzf1*, suppresses the expression of NOTCH target genes during thymocyte differentiation [14,15], whereas PTEN is involved in thymocyte differentiation by controlling the PI3K-AKT-mTOR signaling pathway, which regulates thymocyte survival and proliferation [16,17]. These results indicate that signaling pathways involved in carcinogenesis may differ depending on the age at which an animal is exposed to radiation.

It is well established that the development of thymic lymphoma requires the transformation of bone marrow-derived thymic progenitor cells within the thymus [18,19], and thymectomy reduces the incidence of radiation-induced lymphoma in C57BL mice [20]. Based on transplantation analyses, prelymphoma cells, which have been considered as the origin of radiation-induced thymic lymphomas, develop and expand in the thymus within two weeks following the end of weekly irradiation for four consecutive weeks [21]. 

To examine age-dependent differences in the thymus response during the early period following the end of radiation exposure leading to carcinogenesis, we analyzed cell dynamics during thymic regeneration within three weeks after four consecutive weeks of weekly irradiation starting at age 1 week or 8 weeks in B6C3F1 mice. Our results demonstrate that the dynamics of thymocytes up to three weeks post-irradiation depend on the age at exposure. For irradiated infants in particular, the number of cells increased dramatically, and this correlated with activation of the PI3K-AKT-mTOR pathway. 

## 2. Materials and Methods

### 2.1. Irradiation of Mice and Sample Preparation

Female B6C3F1/Crlj mice were purchased from Charles River Laboratories (Kanagawa, Japan). The mice were housed and irradiated as described previously with minor modification [13]. Briefly, the mice were exposed to weekly whole-body irradiation of 1.2 Gy X-ray for four consecutive weeks starting at age 1 week (irradiated infants) or 8 weeks (irradiated adults) using an X-ray generator (PANTAK Ltd., East Haven, CT, USA) at 0.6 Gy/min. Mice were sacrificed, and the thymus and femur were harvested from nonirradiated mice (age 1–11 weeks), mice at 7 days after the third irradiation, and from 1 to 21 days after the fourth irradiation (Figure S1). At each time point, 6 to 15 mice were studied. The thymus from each mouse was divided into two parts, one of which was fixed for histological analysis and the other was used for making a thymocyte single-cell suspension. The femur was fixed for histological analysis to correlate cellularity and regeneration in these sites with the thymus dynamics after irradiation. All experiments with mice were conducted according to the principles and procedures outlined in our institutional protocols after authorization by the Institutional Animal Care and Use Committee (approval number: 13-1014-3).

### 2.2. Preparation of Thymocytes for Flow Cytometry Analysis

Single-cell suspensions of freshly dissected thymi were obtained by mechanical dissociation with a glass stopper on a 70 µm stainless-steel mesh. The thymocytes were washed with phosphate-buffered saline containing 1% fetal bovine serum, and thymocytes were counted using a hemocytometer. The weight of the thymus used for the preparation of single cell suspension was measured. The cell count, multiplied by the total weight of the thymus, was then divided by the weight of the thymus used for the cell suspension, to normalize the thymocyte number. The suspended single-cell thymocytes were divided into multiple tubes, fixed and frozen with 1% formaldehyde, and then stored at −80 °C. Stored thymocytes were thawed at 37 °C for 3 min and used for flow cytometry analysis within three months after fixation. We confirmed that the majority of the cells in the thymus were Thy1.2 positive at each stage of differentiation following irradiation in our preliminary experiments (data not shown).

### 2.3. Flow Cytometry

Fixed cells (2 × 10^5^ cells) were incubated with antibodies in phosphate-buffered saline containing 1% fetal bovine serum for 30 min or overnight at 4 °C. To detect differentiation markers, the antibodies used were FITC- or phycoerythrin-conjugated anti-CD4 (RM4-5; eBioscience, San Diego, CA, USA) and FITC- or Pacific Blue-conjugated anti-CD8 (53-6.7; eBioscience or BD Bioscience, Franklin Lakes, NJ, USA). Antibodies for CD4 and CD8 were diluted 1:70. To assess activation of the PI3K-AKT-mTOR signaling pathway, FITC-conjugated anti-phospho-AKT (Ser473) (#4071, Cell Signaling Technology, Danvers, MA, USA) was used at 1:7 dilution after incubation for 10–15 min in 100% iced methanol or saponin for permeabilization. To determine the cell-cycle distribution of thymocyte subpopulations, surface-stained cells were resuspended in phosphate-buffered saline containing 10 µM DRAQ5 (eBioscience, San Diego, CA, USA) and incubated for 30 min at 37 °C in a BioShaker (BR-12FM, TAITEC, Saitama, Japan) in the dark. The cell-cycle distribution was determined with a Guava easyCyte flow cytometer (Millipore, Burlington, MA, USA) and analyzed with InCyte and FlowJo software (Tree Star, Ashland, OR, USA). The cell cycle distribution was divided into the G0/G1, S, and G2/M fractions based on the DNA content of singlet cells, with the exception of the sub-G1 peak containing dead cells. Fifty-thousand cells were analyzed for each run.

### 2.4. Immunohistochemistry

For thymus immunohistochemistry, serial 1 µm-thick paraffin sections were used to determine the expression of PI3K (p110α), phospho-AKT (Thr308 and Ser473), AKT (pan), phospho-mTOR (Ser2448), and mTOR. The sections were dewaxed in xylene and rehydrated using a graded ethanol series and heated at 110 °C for 20 min in Tris-EDTA Buffer (10 mM Tris Base, 1 mM EDTA solution, pH 9.0) using a laboratory pressure vessel (Decloaking Chamber NxGen, Biocare Medical, Pacheco, CA, USA) or at 99 °C for 20 min using a microwave. Sections were then incubated overnight at 4 °C with the following primary antibodies at the indicated dilution: anti-PI3 Kinase p110α (C73F8; #4249, Cell Signaling Technology, Danvers, MA, USA) (1:100), anti-phospho-Akt (Thr308) (#9275, Cell Signaling Technology, Danvers, MA, USA) (1:100), anti-phospho-Akt (Ser473) (736E11; #3787, Cell Signaling Technology, Danvers, MA, USA) (1:50), anti-Akt (pan) (11E7; #4685, Cell Signaling Technology, Danvers, MA, USA) (1:400), anti-phospho-mTOR (Ser2448) (49F9; #2976, Cell Signaling Technology, Danvers, MA, USA) (1:100), anti-mTOR (7C10; #2983, Cell Signaling Technology, Danvers, MA, USA) (1:100). After the primary antibodies were washed away, the sections were incubated with a peroxidase-conjugated secondary antibody (Histofine Simple Stain MAX PO(R); Nichirei Biosciences, Tokyo, Japan). Peroxidase activity was visualized by first staining with 3,3′-diaminobenzidine (DAB Peroxidase Substrate Kit (SK-4100), Vector Laboratory, Burlingame, CA, USA) and then counterstaining with hematoxylin. 

### 2.5. Histological Analysis of Thymus and Femur

The remaining thymus and femur were fixed in 10% neutral buffered formalin. Each femur was decalcified following fixation. These tissues were embedded in paraffin, cut in 1 μm sections, and stained with hematoxylin and eosin for histology. Histology slides were scanned using the NanoZoomer-XR slide scanner (Hamamatsu Photonics, Shizuoka, Japan), and the data were stored in a J-SHARE archive (Japan Storehouse of Animal Radiobiology Experiments), which is an animal-experiment archiving system constructed in our institute [22]. NanoZoomer data were analyzed using NDP.view2 Plus viewing software (Hamamatsu Photonics, Shizuoka, Japan). The cellularity in the femur was considered from the proportion of total nucleated cells and scored into five categories compared with the cellularity of normal femur as follows: score −4, 0–20% total nucleated cells; score −3, 20–40%; score −2, 40–60%; score −1, 60–80%; score 0, 80–100%. After irradiation, structural changes in the thymic cortex and medulla were also evaluated.

### 2.6. Statistical Analysis

The results are presented as the mean ± standard error. The data were analyzed using Prism 8.0 software (GraphPad Inc., San Diego, CA, USA). Statistical differences between two sets of independent data were determined using the Student’s or Welch’s *t*-test based on the results of the *F*-test. Differences with *p* < 0.05 were considered statistically significant.

## 3. Results

### 3.1. Post-Irradiation Cell Dynamics during Thymic Regeneration Differ Depending on Age at Exposure

**Figure 1:**
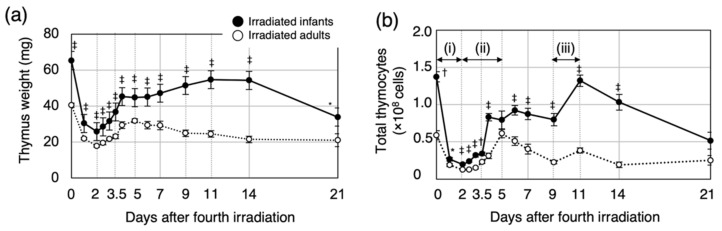
Change in thymus weight and cell number after whole-body irradiation of infant and adult mice. (**a**) Change in thymus weight of B6C3F1 mice after irradiation at infancy or adulthood. (**b**) Change in thymocyte number after the fourth irradiation. The recovery of thymocyte numbers was classified into three phases: reduction phase (i), first regeneration (ii), and second regeneration (iii). Six to fifteen mice were analyzed for each time point. Closed circles, average for irradiated infant mice; open circles, average for irradiated adult mice. * *p* < 0.05; ^†^ *p* < 0.01; ^‡^ *p* < 0.001, as compared with irradiated adult mice. Thymus weight and thymocyte counts in age-matched non-irradiated mice are shown in Figure S6.

To investigate the age-dependent dynamics in the regenerating thymus after irradiation, we first compared thymus weight and total number of thymocytes in irradiated infants with those in irradiated adults (Figure 1). For both irradiated groups, the thymus weight decreased after the last irradiation and reached a minimum at 2 days post-irradiation (d2, Figure 1a). The thymus weight then increased until d4. For the irradiated infants, the thymus weight increased gradually from d6 to d11 and then decreased between d14 and d21. On the other hand, for the irradiated adults, the thymus weight gradually decreased after d5. Thymus weight for the irradiated infants was significantly greater than that of irradiated adults at all time points until d21.

The dynamics of the thymocyte number consisted of three phases (Figure 1b): a reduction phase (i) and the first (ii) and second regeneration (iii) phases. The reduction phase continued up to d2. For both groups, the thymocyte number decreased by approximately 80% at d1, and this decrease was accentuated at d2. The first regeneration phase occurred between d2 and d5. For irradiated infants, the thymocyte number increased markedly between d3.5 and d4 and then remained constant until the second regeneration phase. On the other hand, for the irradiated adults, the thymocyte number increased markedly between d4 and d5 and then decreased gradually up to the second regeneration phase. The first regeneration phase occurred sooner for the irradiated infants than the irradiated adults. For both groups, the second regeneration phase occurred between d9 and d11. For the irradiated infants, the thymocyte number increased markedly and then decreased steadily to d21. For the irradiated adults, the thymocyte number increased slightly and remained constant after the second regeneration phase. At all time points except d5 and d21, the thymocyte number was significantly greater for the irradiated infant mice than for the irradiated adults. These results suggested that the dynamics of thymic regeneration after irradiation depend on age at exposure.

### 3.2. Proliferation of Immature Thymocytes Plays a Key Role in Thymic Regeneration in Mice Irradiated during Infancy

**Figure 2:**
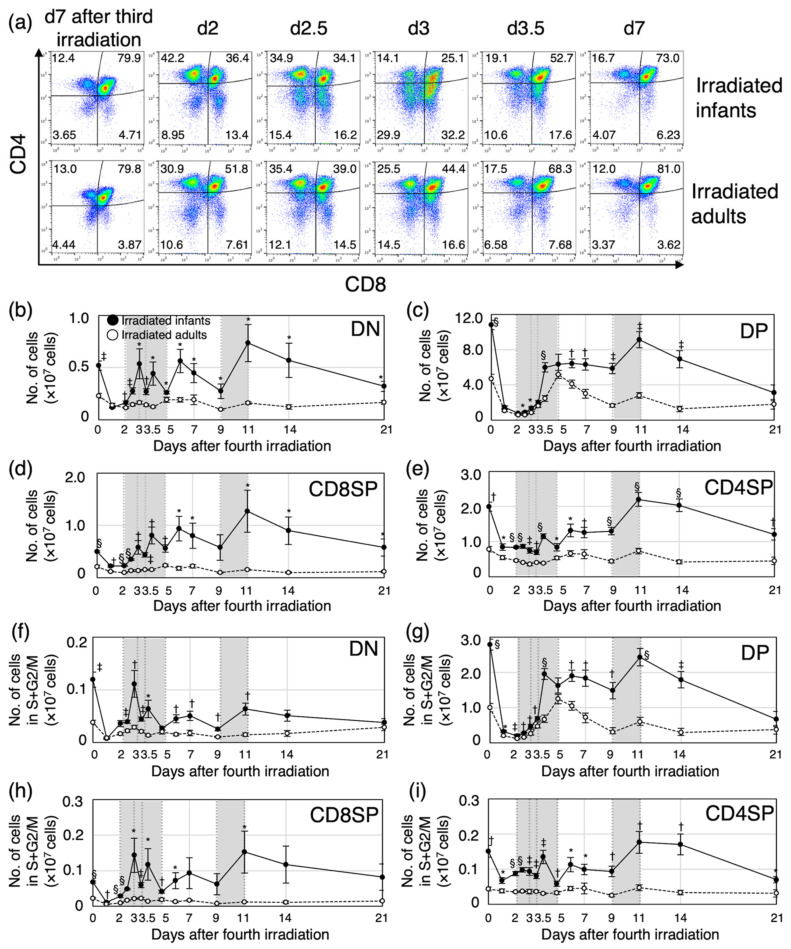
Dynamics of four differentiation stages with regard to the proportion and number of thymocytes after the fourth irradiation. (**a**) Representative plots for CD4 and CD8 expression in thymocytes from irradiated infant or adult mice during the first regeneration (from d7 after the third irradiation to d7 after the fourth irradiation). (**b**–**e**) The number of cells in each population was calculated from the proportion and the total number of thymocytes from mice sacrificed 7 days after the third irradiation and from 1 day to 3 weeks after the fourth irradiation. (**f**–**i**) The number of cells in each cell-cycle phase was calculated from the proportion and the total number of thymocytes. Six to fifteen mice were analyzed for each time point. (**b**,**f**) CD4 and CD8 double negative (DN). (**c**,**g**) CD4 and CD8 double positive (DP). (**d**,**h**) CD8 single positive (SP). (**e**,**i**) CD4SP. Closed circles, irradiated infant mice; open circles, irradiated adult mice. Gray squares in each graph show the first or second regeneration period. * *p* < 0.05; ^†^ *p* < 0.01; ^‡^ *p* < 0.001; ^§^ *p* < 0.0001, as compared with irradiated adult mice.

To gain a deeper understanding of the mechanism of age-dependent cell dynamics during thymic regeneration after irradiation, we next analyzed the cell-cycle phases during the four differentiation stages and determined both the relative proportion and absolute number of thymocytes in phases S, G2 and M (hereafter S+G2/M, i.e., cycling cells). Thymocyte differentiation steps are defined according to the expression of major cell-surface markers, CD4 and CD8 (Figure S2). The most immature thymocytes express neither CD4 nor CD8 (CD4^−^CD8^−^, double-negative, DN). After β-selection, DN thymocytes progress through the rapidly cycling CD8^+^TCR-αβ^−^ immature single-positive stage to yield CD4^+^CD8^+^ double-positive (DP) cells. DP thymocytes express TCR-αβ and undergo both positive and negative selection to yield TCR-αβ^+^CD4^+^ and TCR-αβ^+^CD8^+^ single-positive (SP) thymocytes. Because we did not measure the expression of TCR-αβ, the CD8SP population in our study included both immature and mature cells. Cell-surface expression of CD4 and CD8 was assessed by flow cytometry (Figure 2a). The proportion of DP thymocytes decreased from 80% of total thymocytes (approximately the same proportion as in normal thymus) to 25% (irradiated infants) and 40% (irradiated adults) until d3. For both irradiated groups, the proportion of DP thymocytes returned to the normal level up to d7. Interestingly, there was marked expansion of the DN and CD8SP populations (these included immature cells) between d2 and d3 for the irradiated infants, yet little expansion of these two populations was observed for the irradiated adults. The number of cells in each population was calculated based on the proportion and the total number of cells (Figure 2b–e). The number of cells in each of the four populations decreased up to d2 for each of the irradiated groups. Corresponding to the change in the proportion (Figure 2a), the number of DN and CD8SP thymocytes in irradiated infants increased markedly between d2 and d3, whereas the increase was marginal for the irradiated adults (Figure 2b,d). The number of DP thymocytes increased between d2.5 and d4 in irradiated infants and between d3 and d5 in irradiated adults (Figure 2c). The maximum specific growth rate per day [23] of DP thymocytes was 2.17 between d3.5 and d4 in irradiated infants, and 1.26 between d3 and d3.5 in irradiated adults, suggesting that cell proliferation activity during the first regeneration phase might be higher in irradiated infants than in irradiated adults. In both groups, the number of mature CD8SP and CD4SP thymocytes tended to increase in conjunction with the increase in DP thymocytes (Figure 2d,e). After the first regeneration phase, the number of cells at all four differentiated stages remained essentially constant up to d9, except for DP thymocytes, which decrease in irradiated adults. Between d9 and d11, there was a peak in the number of cells undergoing the second regeneration phase in both irradiated groups, although the peak was higher for the irradiated infants, especially for DN and CD8SP thymocytes (Figure 2b–e). After the second regeneration phase, the number of thymocytes in the four differentiation stages tended to decrease over time in the irradiated infants, whereas the number remained constant in the irradiated adults.

To determine whether there was an association between the number of post-regeneration cells and cell proliferation rate in each of the irradiated-infant and -adult experimental groups, we also measured the proportion of cycling cells, i.e., S+G2/M, in each thymocyte subpopulation based on the DNA content (Figure S3). The number of cycling cells was significantly greater in the irradiated infant mice than in the irradiated adults, reflecting a marked increase in cell proliferation in the infants during thymic regeneration (Figure 2f–i).

Taken together, these data suggested that the increase in the proportion of proliferating immature thymocytes is the key response to radiation exposure during the first thymic regeneration, and the potential for regeneration may depend on the number of surviving and proliferating thymocytes after irradiation.

### 3.3. The Impact of the PI3K-AKT-mTOR Pathway on Cell Proliferation and Cell Survival Varies by Age at Exposure and with the Phase of Post-Exposure Thymic Regeneration

**Figure 3:**
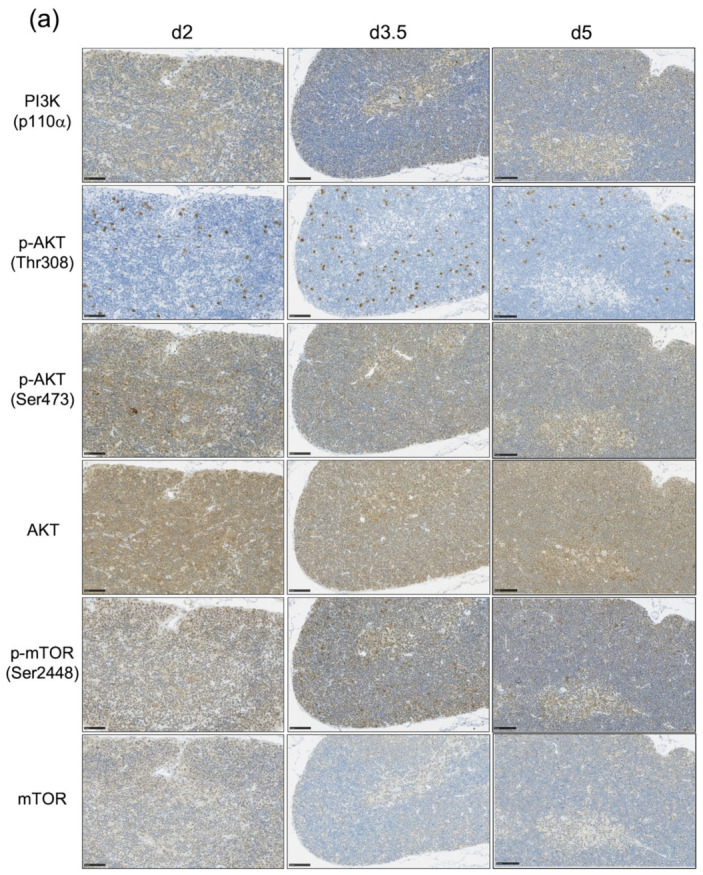

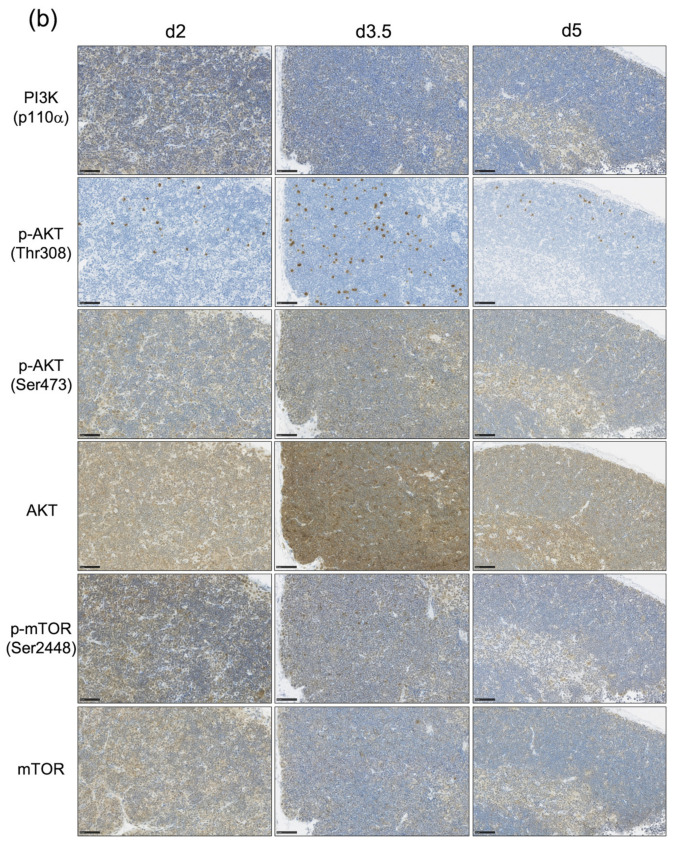
Immunohistochemical analysis of the expression of the PI3K-AKT-mTOR pathway-associated proteins during the first regeneration. The expression of PI3K (p110α), phospho-AKT (Thr308 and Ser473), AKT (pan), phospho-mTOR (Ser2448), and mTOR were analyzed to assess the activation of the PI3K-AKT-mTOR pathway in the thymus during the first regeneration phase. At least three immunostained images were acquired at the same time point, and representative images are shown. (**a**) Irradiated infants; (**b**) irradiated adults. All sections were counterstained with hematoxylin. Scale bar in all panels represents 50 µm.

Our previous study suggested that *Pten* mutated cells, in which the activation of the PI3K-AKT-mTOR pathway is thought to be dysregulated, might be the candidate cell of origin for lymphomagenesis in irradiated infants. Thus, we hypothesized that the proportion of thymocytes in which the PI3K-AKT-mTOR pathway is activated might be greater for irradiated infant mice than irradiated adults. To assess the involvement of the PI3K-AKT-mTOR pathway in the thymic regeneration, we next examined the expression of six representative proteins associated with the activation of this pathway using immunohistochemistry (Figure 3 and Figure S4). The results showed that these three phosphorylated proteins, i.e., p-AKT (Thr308), p-AKT (Ser473), and p-mTOR (Ser2448), tended to be highly expressed at d2, when the first regeneration begins, increased at d3.5, and then decreased at d5, when the first regeneration ends. Interestingly, the PI3K (p110α), p-AKT (Ser473) and p-mTOR (Ser2448)-positive cells were located in the same place in serial sections, i.e., they were abundant in the subcapsular cortex and medulla (Figure 3 and Figure S4). The proportion of cells positive for the three phosphorylated proteins at each time point appeared to be higher in irradiated infants than irradiated adults (Figure 3).

**Figure 4:**
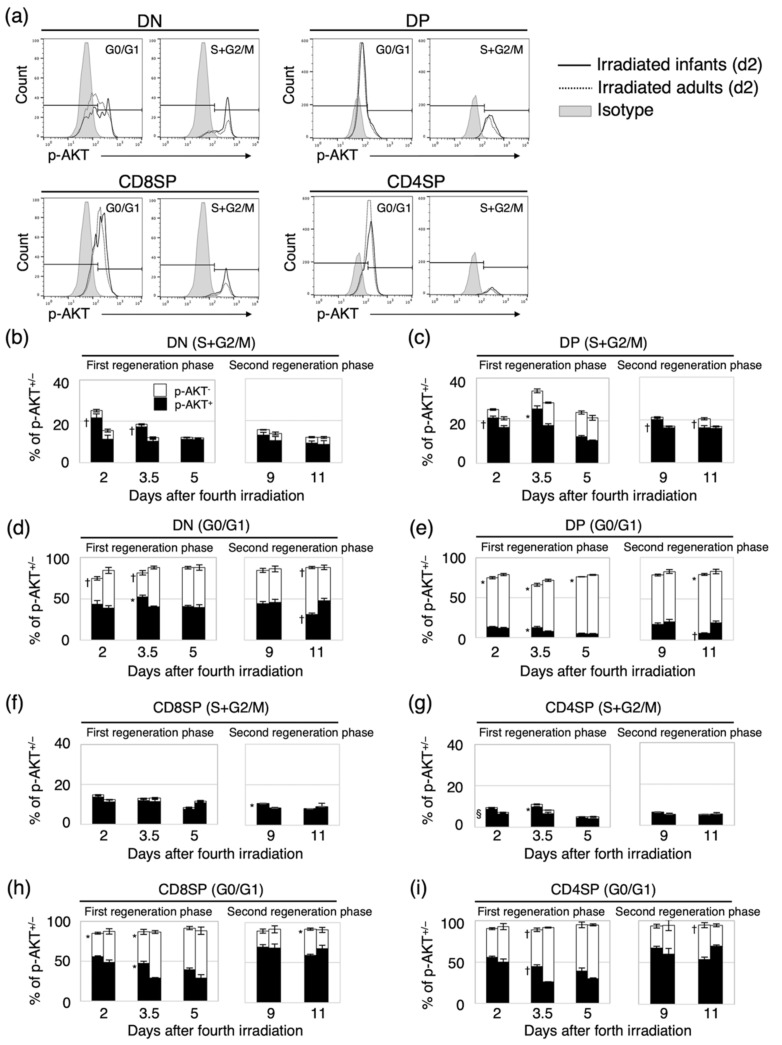
Change in the proportion of phosphorylated-AKT-positive (p-AKT^+^) cycling cells in the four differentiation stages in each of the two regeneration phases. (**a**) Representative histograms of flow cytometry experiments for quantifying p-AKT^+/−^ cells in cell-cycle phases G0/G1 and S+G2/M in the four differentiation stages. The results at d2 after the fourth irradiation are shown. (**b**–**i**) Change in the proportion of p-AKT^+/−^ thymocytes in the four differentiation stages during each of the two regeneration phases. (**b**,**d**) CD4 and CD8 double negative (DN); (**c**,**e**) CD4 and CD8 double positive (DP); (**f**,**h**) CD8 single positive (CD8SP); (**g**,**i**) CD4SP. Shown is the proportion of p-AKT^+^ or p-AKT^−^ cells in S+G2/M and G0/G1 in each differentiation stage. The data were acquired during the two thymic regeneration phases after irradiation. A subset of the mice used for experiments presented in Figure 2 was analyzed (three to eight mice for each time point). Left bars, irradiated infant mice; right bars, irradiated adult mice. Black bars, p-AKT^+^ cells; white bars, p-AKT^−^ cells. * *p* < 0.05; ^†^ *p* < 0.01; ^§^ *p* < 0.0001, as compared with the corresponding proportion of p-AKT^+^ cells in irradiated adult mice.

The activation of the PI3K-AKT-mTOR pathway in thymocytes is required for their survival and proliferation [16]. Both AKT activation and *PTEN* deletion are also associated with cell-cycle progression in embryonic stem cells and tumor cells [24,25]. The activation of the PI3K-AKT-mTOR pathway was confirmed by immunohistochemistry (Figure 3 and Figure S4). We could not quantify the difference in the distinct differentiation stages and the dependence in the age at exposure. Thus, the cell-cycle distribution (S+G2/M or G0/G1) and the proportion of p-AKT-positive (p-AKT^+^) cells in the four differentiation stages of thymocytes were analyzed to quantify the age-dependent involvement of the PI3K-AKT-mTOR pathway in thymic regeneration after irradiation in each differentiation stage (Figure 4). 

During the first regeneration phase (between d2 and d5), the proportion of p-AKT^+^ thymocytes in S+G2/M in the DN and DP populations at d2 and d3.5 in irradiated infants was significantly greater than that in irradiated adults but decreased thereafter to the same level as for irradiated adults at d5 (Figure 4b,c, left panel). At d3.5, the proportion of p-AKT^+^ cells in G0/G1 in the DN and DP populations was also significantly greater in the irradiated infants than in the irradiated adults (Figure 4d,e, left panels). These results indicated that, in these two populations, the proportions of cycling cells and surviving cells in which the PI3K-AKT-mTOR pathway was activated were greater in irradiated infants than in irradiated adults. For the CD8SP population, although there was no age-dependent difference in the proportion of p-AKT^+^ cells in S+G2/M (Figure 4f, left panel), the proportion of p-AKT^+^ cells in G0/G1 at d3.5 was significantly greater in irradiated infants than in irradiated adults (Figure 4h, left panel). For the CD4SP population, the proportion of p-AKT^+^ cells in S+G2/M was significantly greater in irradiated infants than in irradiated adults, but the difference in the proportion between the two groups was small compared with that of the DN and DP populations (Figure 4g, left panel). The proportion of p-AKT^+^ cells in G0/G1 in the CD4SP population had the same tendency as that in the CD8SP population, and at d3.5, the difference between the two groups was significant (Figure 4i, left panel). These results indicated that, in mature CD8SP and CD4SP thymocytes, activation of the PI3K-AKT-mTOR pathway during G0/G1 was more prevalent in irradiated infants than in irradiated adults. Altogether, during the first thymic regeneration, the activation of the PI3K-AKT-mTOR pathway was involved in not only the proliferation and survival of immature DN and DP thymocytes but also the survival of mature CD8SP and CD4SP thymocytes in irradiated infants more so than in irradiated adults. These age at exposure-dependent differences may have resulted in marked increases in the number of cells in irradiated infants compared with irradiated adults.

During the second regeneration phase (between d9 and d11), the proportion of p-AKT^+^ cells in S+G2/M at d9 was greater for irradiated infants than irradiated adults in all four thymocyte populations, and the difference was statistically significant for the DP and CD8SP populations (Figure 4c,f, right panels). In the DN population, the proportion of p-AKT^+^ cells in S+G2/M at d9 was smaller than that at d2, but unexpectedly, the number of cells was significantly greater at d11 (Figure 2b and Figure 4b). Compared with d2, however, the proportion of p-AKT^+^ cells in G0/G1 at d9 was the same in the DN population but greater in the DP, CD8SP, and CD4SP populations (Figure 4d,e,h,i). These results suggested that the activation of the PI3K-AKT-mTOR pathway may be more involved in cell survival than proliferation during the second regeneration compared with the first regeneration.

Interestingly, at d11, the proportion of p-AKT^+^ cells in G0/G1 in all four thymocyte populations was greater for the irradiated adults than the infants (Figure 4d,e,h,i), right panels). The fact that a decrease in the proportion of thymocytes in which the PI3K-AKT-mTOR pathway became activated was associated with a decrease in the number of thymocytes after d11 in irradiated infants (Figure 2b–e) suggested that the activation of this signaling pathway is required for thymocyte survival.

### 3.4. Bone-Marrow Injury after the Fourth Irradiation Is More Severe in Irradiated Infants Than in Irradiated Adults

**Figure 5:**
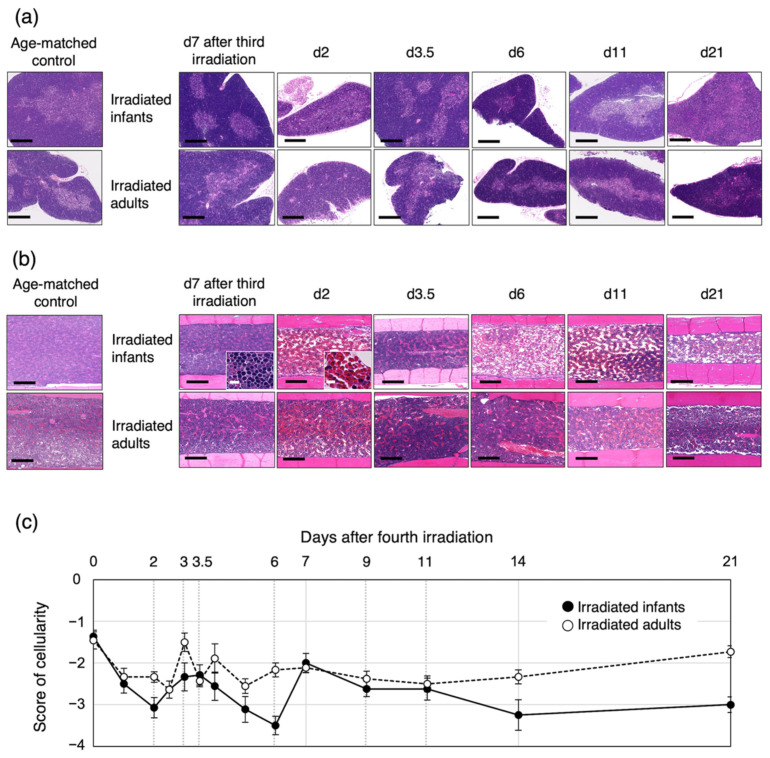
Histological analysis of thymus and femur by hematoxylin and eosin staining in mice irradiated during infancy or adulthood. Representative images of mouse thymus (**a**) and femur (**b**) at 7 days after the third irradiation or at 2, 3.5, 6, 11 or 21 days after the fourth irradiation. Scale bar in all panels represents 250 µm (10 µm in each inset). Thymus and femur from mice age at 4 and 11 weeks are shown as age-matched control for the irradiated infants and adults, respectively. The structure of thymic cortex and medulla was completely destroyed at d2. Nucleated cells in bone marrow were almost depleted, and red blood cells were prominent in bone marrow at d2. (**c**) Scoring of the proportion of nucleated cells in bone marrow. Six to fifteen mice were analyzed for each time point. Closed circles, irradiated infant group; open circles, irradiated adult group.

Because thymocyte progenitor cells are derived from bone marrow, we performed a histological analysis of the thymus and femur after the fourth irradiation (Figure 5). In both irradiated infants and adults, the structure of the thymic cortex and medulla had completely collapsed at d2, and then structural reconstitution was observed at d3.5, i.e., during the first thymic regeneration phase (see Figure 1, Figure 2 and Figure 5a). Thereafter, the structure of each of the cortex and medulla within the thymus was maintained, but each gradually collapsed at d21 in the irradiated infants (Figure 5a), when the number of thymocytes had decreased significantly (Figure 1 and Figure 2).

The proportion of nucleated cells in the bone marrow decreased significantly until d2 in both groups and then partially recovered between d2 and d3 (Figure 5b,c). For the irradiated infant mice, the proportion of nucleated cells continued to decrease until d6 and then increased from d6 to d7 to the same level as the irradiated adults (Figure 5c). On the other hand, it fluctuated between d3 and d6 in the irradiated adults. The same proportion was maintained in both irradiation groups between d9 and d11, i.e., during the second thymic regeneration, but the proportion tended to decrease from d14 to d21 in irradiated infants compared with irradiated adults. Up to d21, the cellularity in bone marrow in both groups did not reach the same level as d7 after the third irradiation (Figure 5c). These results suggested that the bone marrow in both irradiated infants and adults could not supply the normal level of precursor cells to the thymus. In particular, we concluded that the bone-marrow cells were more severely damaged in irradiated infants than in irradiated adults; therefore, bone-marrow regeneration might take longer for irradiated infants than irradiated adults.

## 4. Discussion

In this study, we used a mouse model of human acute lymphoblastic leukemia to analyze the cell dynamics of post-irradiation thymic regeneration, during which prelymphoma cells develop. The results revealed that the activation of the PI3K-AKT-mTOR pathway contributed to thymic regeneration through differential effects on cell survival and/or proliferation during the two regeneration phases in irradiated infants. Furthermore, the cell dynamics during thymic regeneration after irradiation differed depending on the age at exposure, and this difference may have affected the selection of the cells of origin for radiation-induced mouse thymic lymphoma.

In the present study, the first regeneration began earlier for irradiated infants than for adults, and regeneration capacity was likewise greater for infants (Figure 1 and Figure 2). Previous bone-marrow transplantation experiments demonstrated that progenitor cells differentiate into DP 13 days after the transfer of progenitor cells to the thymus [26]. This report indicates that the first regeneration of the thymus observed in the present study was caused by the proliferation of surviving immature thymocytes. In fact, the thymic regeneration capacity decreased with age (between ages 6 and 17 months) in relation to the number of DN1 cells in the thymus [27]. Therefore, the regeneration capacity may depend on the number of immature cells that have the potential to proliferate, which may be the mechanism underlying the observed age at exposure-dependent and radiation dose-dependent thymic regeneration. On the other hand, during the second regeneration phase, the number of immature thymocytes increased without an increase in the proportion of S+G2/M cells (Figure 2 and Figure S5). The proportion of nucleated cells in the bone marrow increased from d2 after the fourth irradiation (Figure 5). This suggests that, unlike the first regeneration, the second regeneration may be a consequence of both an increase in the number of cells transferred from the bone marrow into the thymus and cell differentiation in the thymus, as seen in nonirradiated mice. These results suggest that the first and second regenerations were caused by different mechanisms and that the regeneration capacity is greater at a younger age.

Although the PI3K-AKT-mTOR pathway regulates the survival of DP thymocytes during the normal thymocyte differentiation process [16,28,29], we showed that activation of the pathway promoted proliferation of DP as well as DN thymocytes, especially during the first regeneration in irradiated infants (Figure 4). In nonirradiated mice, the number of p-AKT^+^ thymocytes was greater at a younger age for all differentiation stages and decreased with age (Figure S6). After radiation-induced thymocyte depletion on d2, the proportion of p-AKT^+^ cells in immature populations and in G0/G1 of mature populations was greater for the irradiated infants than the adults (Figure 4b–e). Cytokine-mediated activation of the PI3K-AKT-mTOR pathway is also involved in cellular radioresistance [30]. It has also been reported that AKT-mediated phosphorylation of Niban plays a pivotal role in protecting cells from cell death by promoting the dissociation of MDM2 from nucleophosmin and the subsequent degradation of TP53 [31]. Based on these results, we conclude that the proportion of cells in which the PI3K-AKT-mTOR pathway is activated is greater at a younger age, regardless of the stage of differentiation, and influences the regeneration capacity after thymocyte depletion. 

Although little is known about the functions and mechanisms of regulation in the S-, G2-, and M-phase of the cell cycle, it is well established that PI3K/AKT signaling activation by growth factors regulates the G1 phase of cell cycle progression [32]. In particular, AKT regulates the phosphorylation of p21^Cip1^ and p27^Kip1^, inhibitors of cell cycle progression, and controls the arrest of G1 phase and the progression to S phase, respectively [33,34,35,36]. It is also known to induce Cyclin D accumulation through phosphorylation of GSK-3 [37].

However, this study did not address the possibility of any direct involvement of PTEN in the biphasic regeneration after irradiation. In thymocytes, IL-7R and TCR-mediated signaling is involved in differentiation and regeneration of thymocytes by activating the PI3K-AKT-mTOR pathway [17,38,39]. These results suggest that activation of the pathway during both differentiation and regeneration may be tightly regulated by PTEN, especially in DP thymocytes. Furthermore, CD4SP lymphoma originating from DP thymocytes is frequently observed in T cell-specific *Pten*-deficient mice [40], supporting the idea that *Pten*-deficient cells might be selected as prelymphoma cells during thymic regeneration—as seen in irradiated infants in the present study. These data suggest that *Pten*-deficient cells likely have a proliferation advantage during thymic regeneration after irradiation. This possibility could be addressed via experiments with *Pten*-deficient mice. On the other hand, the frequency of mutations in the *Pten* and *Ikzf1* genes was similar in the thymic lymphomas induced after a single irradiation at infancy [11]. This result suggests that four-fractionated irradiations from infancy, which induce repeated regeneration of the thymus, are strongly associated with activation of the PI3K-AKT-mTOR pathway and may increase the probability that *Pten*-mutated cells are selected as the cells of origin for thymic lymphoma. Our previous study showed that *Pten* abnormalities in thymic lymphomas arising in irradiated infants are associated with chromosomal mis-segregation or mitotic recombination [13]. Chromosomal mis-segregation occurs when duplicated chromosomes separate unequally during cell division [41], and mitotic recombination causes chromosomal exchange during interphase in cycling cells [42,43]. These findings reinforce our hypothesis that *Pten* abnormalities occur in proliferating cells and are involved in lymphomagenesis in irradiated infants. On the other hand, we did not unequivocally establish the cause of the observed high frequency of *Ikzf1* abnormalities with regard to the development of radiation-induced thymic lymphomas. Because abnormalities in *Ikzf1* reportedly induce aberrant thymocyte differentiation [44,45], cells with abnormalities in *Ikzf1* may not require the age at exposure-dependent rapid cell proliferation observed during thymic regeneration and may be selected as the cells of origin for radiation-induced thymic lymphoma. In the future, it will be necessary to investigate when *Ikzf1* abnormalities occur during thymic lymphomagenesis in irradiated adults.

In addition, the thymic regeneration capacity observed in this study is considered to depend not only on the number of p-AKT^+^ cells surviving in the thymus after irradiation but also on signal transduction from the surrounding microenvironment. Unfortunately, we were not able to determine which cytokines activate the PI3K-AKT-mTOR pathway in this study. IL-7/IL-7R signaling is a major pathway that activates the PI3K-AKT-mTOR pathway, which is involved in thymocyte differentiation and proliferation, and in T-ALL development [46,47,48]. It has also been reported that IL-9 activates the PI3K-AKT-mTOR pathway in synovial T cells [49]. However, we previously showed that IL-9 alone did not promote thymocyte proliferation, while in combination with IL-7, it synergistically enhances cell proliferation [46], and may activate the JAK-STAT pathway during thymic lymphoma development [50]. The JAK-STAT pathway may also be involved in thymic regeneration as IL-7 signaling plays a role in the differentiation of normal thymocytes by activating the JAK-STAT pathway as well as the PI3K-AKT-mTOR pathway [38]. Radiation-induced activation of the TRP53 pathway results in apoptosis of DP thymocytes and has been reported to be the mechanism for radiation-induced thymic involution [51]. The depletion of DP thymocytes promotes thymic regeneration owing to IL-23 production by radio-resistant dendritic cells followed by IL-22 production by lymphoid-tissue inducer cells [52]. These results suggest that the rapid depletion of DP thymocytes after irradiation that we also observed in the present study may have induced the cytokine signaling for thymic regeneration. In addition, it has been reported that IL-22 promotes proliferation of both human breast and lung cancer cells through the activation of the PI3K-AKT-mTOR pathway [53,54]. Because the transformation of thymocytes into thymic lymphoma is thought to occur in the thymic microenvironment [18,19], the irradiated thymic microenvironment may affect the thymocyte dynamics. In fact, we previously reported that certain cytokines such as IL-1β, IL-12p40 and IL-11 were secreted from macrophages after irradiation as assessed with a mouse model in which nearly 100% of mice develop thymic lymphoma after four-fractionated irradiation [55]. In particular, IL-11, which is elevated in the human colorectal cancer microenvironment [56], mediates the radioresistance of cervical cancer cells via the PI3K-AKT-mTOR pathway [57]. These results suggest that the irradiated thymic microenvironment is involved in thymic carcinogenesis through the activation of PI3K-AKT-mTOR signaling via the expression of certain cytokines, but the effect(s) of age at exposure on the thymic microenvironment needs further investigation.

## 5. Conclusions

This study demonstrates that cell dynamics in the biphasic regeneration of the thymus after irradiation differ with age at exposure. The results also show that cell proliferation and cell survival through activation of the PI3K-AKT-mTOR pathway might have a role in mouse thymic regeneration after irradiation, depending on age at exposure and regeneration phases. These observations will contribute to the elucidation of the risk of thymic lymphomagenesis depending on age at exposure, but further investigation might be needed to reveal the overall mechanism.

## Data Availability

The data presented in this study are available on request from the corresponding author. The data are not publicly available because the conclusion of the collaborative research agreement with QST-NIRS is required in the use of data.

## Supplementary Materials

The following supporting information can be downloaded at: https://www.mdpi.com/article/10.3390/biology11030449/s1, Figure S1: Experimental design for the thymic regeneration analysis, Figure S2: Thymocyte differentiation process based on the expression of CD4 and CD8 on the cell surface., Figure S3: Representative flow cytometry profiles for the cell-cycle analysis during thymic regeneration in the four differentiation stages, Figure S4: Immunohistochemical analysis of the expression of the PI3K-AKT-mTOR pathway-associated proteins during the second regeneration phase, Figure S5: Change in the proportion of cells in cell cycle phase S+G2/M in each differentiation stage after the fourth irradiation, Figure S6: Change in the number of cells and the proportion of phosphorylated-AKT positive (p-AKT^+^) thymocytes in nonirradiated mice at different ages.
