# Supplementary material for: Post-Irradiation Thymic Regeneration in B6C3F1 Mice Is Age Dependent and Modulated by Activation of the PI3K-AKT-mTOR Pathway
Source: Biology (Basel). 2022 Mar 16;11(3):449. doi: 10.3390/biology11030449 (PMC8945464; doi:10.3390/biology11030449)
Supplement: Supplementary file 1 [file biology-11-00449-s001.zip › biology-1535307-supplementary.pdf]

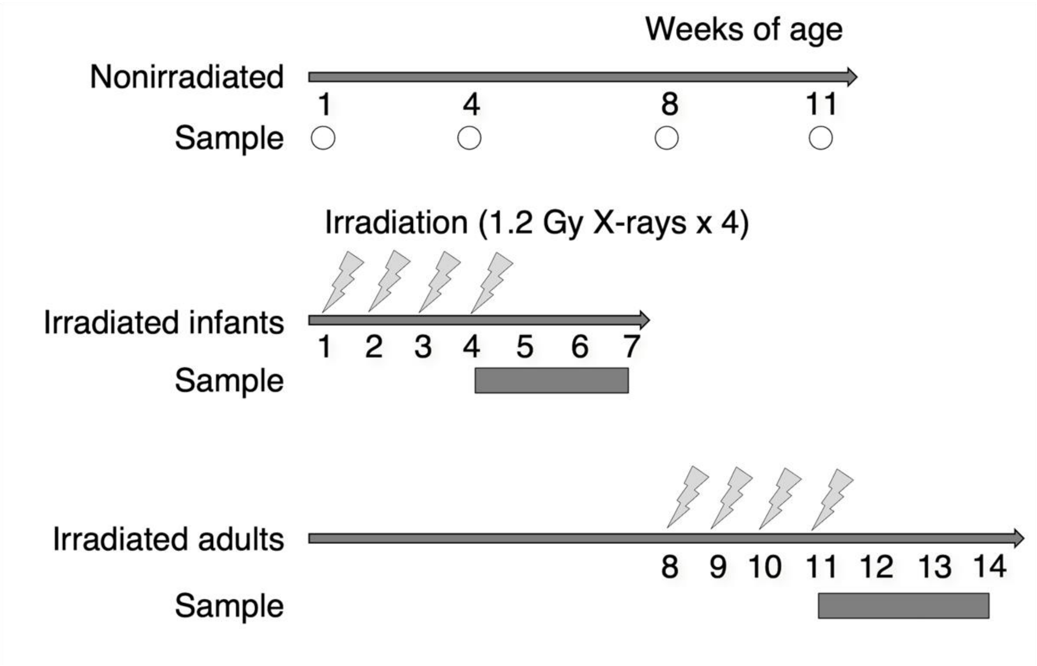

Figure S1. Experimental design for the thymic regeneration analysis. Nonirradiated mice were sacrificed at age 1, 4, 8 and 11 weeks, shown as open circles. Female B6C3F1/Crlj mice were exposed to 1.2 Gy whole-body X-rays for four consecutive weeks starting at age 1 week (infancy) or 8 weeks (adulthood). In the irradiation groups, mice were sacrificed at 7 days after the third irradiation or at 1, 2, 2.5, 3, 3.5, 4, 5, 7, 9, 11, 14 or 21 days after the fourth irradiation, shown as gray bars. A total of 6 to 15 mice were analyzed for each time point.

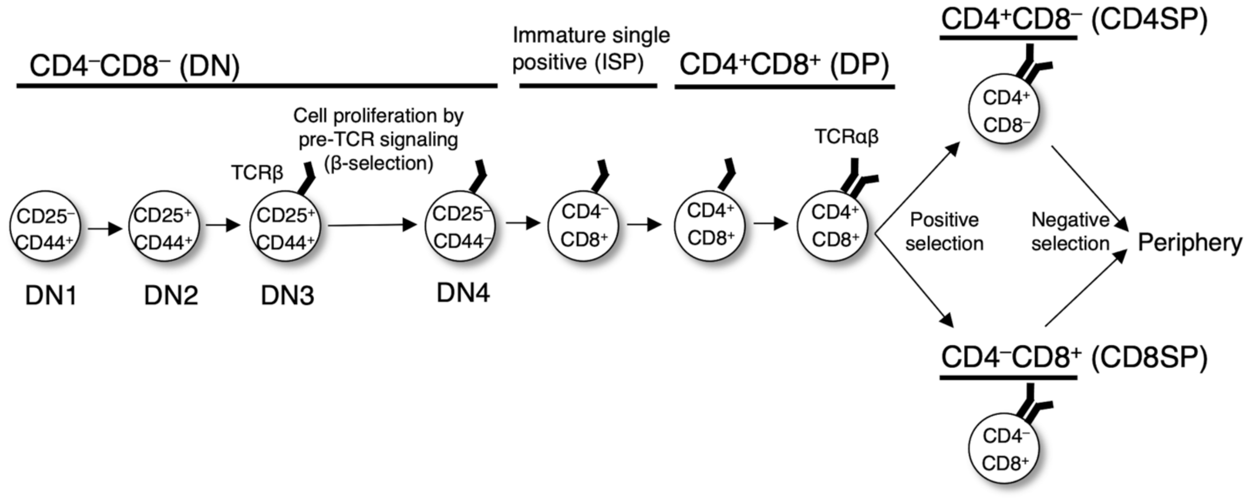

Figure S2. Thymocyte differentiation process based on the expression of CD4 and CD8 on the cell surface. Thymocyte differentiation steps are defined according to the expression of the two major cell-surface markers, namely CD4 and CD8. The most immature thymocytes express neither CD4 nor CD8 ($CD4^{-}CD8^{-}$, double-negative, DN). After β-selection, DN thymocytes progress through the rapidly cycling $CD8^{+}TCR\text{-}\alpha\beta^{-}$ immature single-positive stage to yield $CD4^{+}CD8^{+}$ double-positive (DP) cells. DP thymocytes express TCR-αβ and undergo both positive and negative selection to yield $TCR\text{-}\alpha\beta^{+}CD4^{+}$ and $TCR\text{-}\alpha\beta^{+}CD8^{+}$ single-positive (SP) thymocytes.

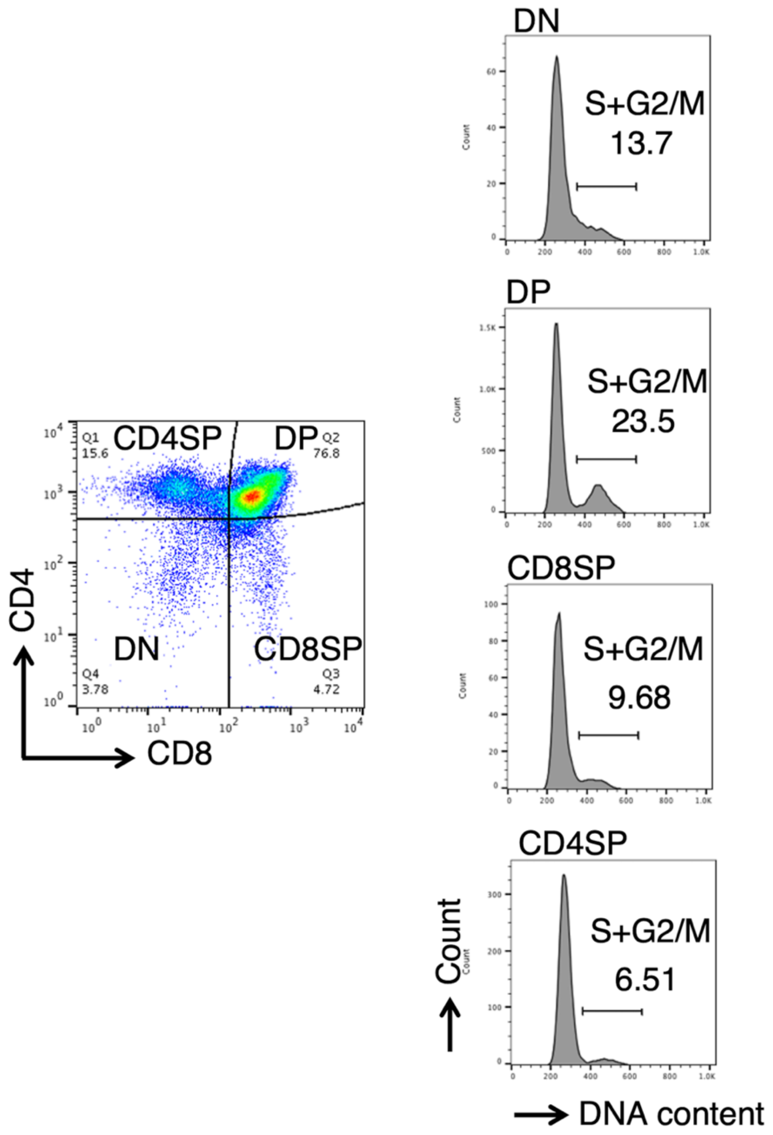

Figure S3. Representative flow cytometry profiles for the cell-cycle analysis during thymic regeneration in the four differentiation stages. Left panel, representative plots for CD4 and CD8 expression in thymocytes. Right panel, representative profiles for cell-cycle distribution based on DNA content.

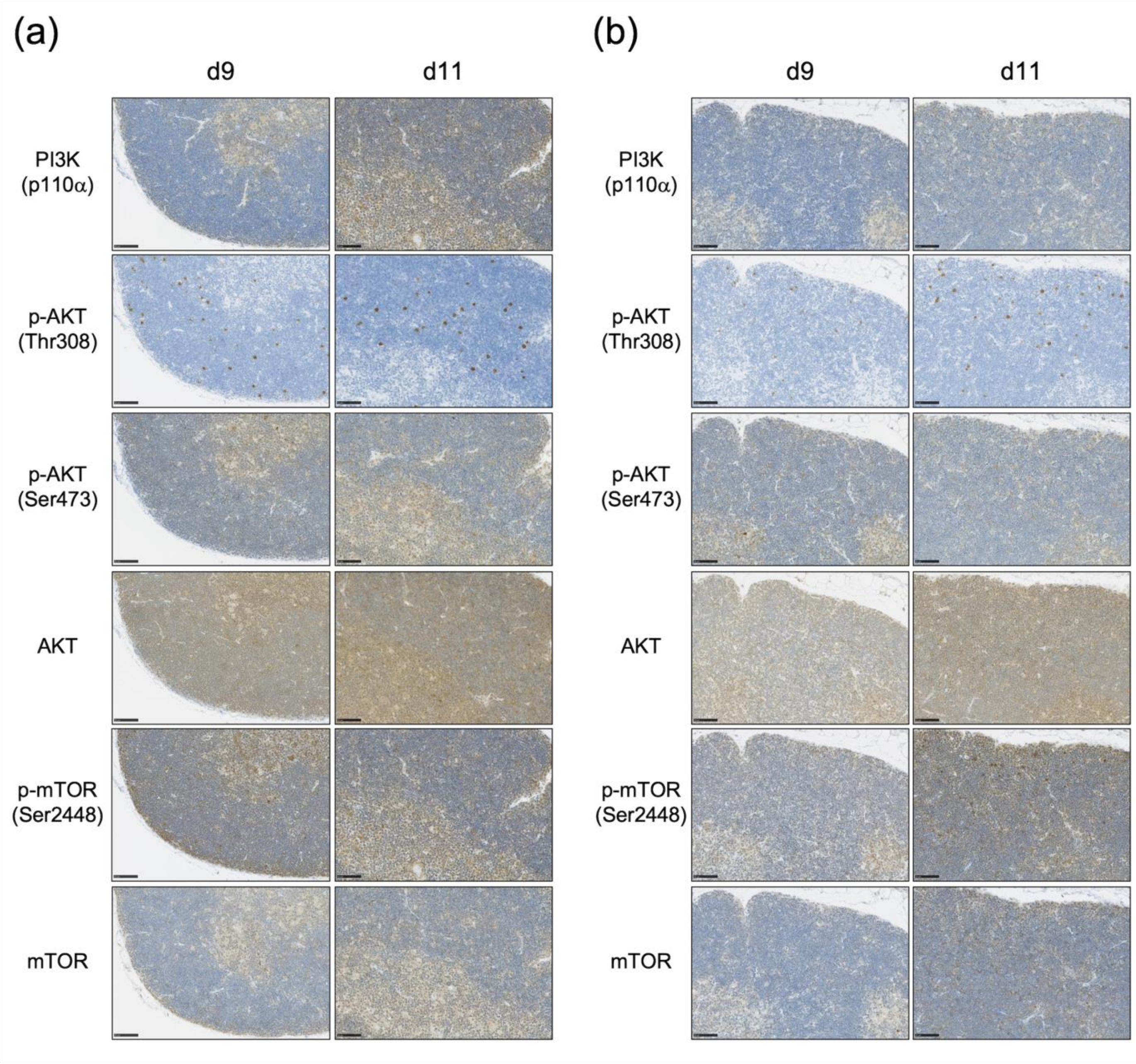

Figure S4. Immunohistochemical analysis of the expression of the PI3K-AKT-mTOR pathway–associated proteins during the second regeneration phase. The expression of PI3K (p110α), phospho-AKT (Thr308 and Ser473), AKT (pan), phospho-mTOR (Ser2448), and mTOR were analyzed to assess the activation of the PI3K-AKT-mTOR pathway in the thymus during the second regeneration phase. At least three immunostained images were acquired at the same time point, and representative images are shown. (a), irradiated infants; (b), irradiated adults. All sections were counterstained with hematoxylin. Scale bar in all panels represents 50 μm.

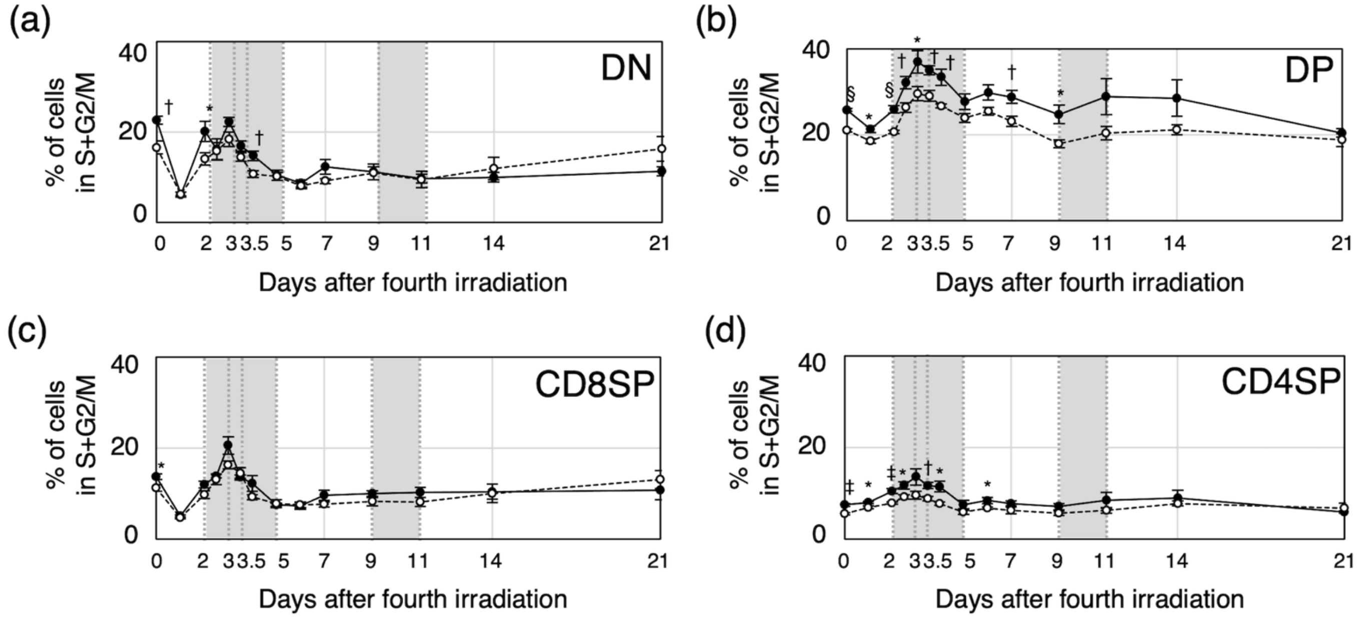

Figure S5. Change in the proportion of cells in cell cycle phase S+G2/M in each differentiation stage after the fourth irradiation. (a) CD4- and CD8-double negative (DN). (b) CD4- and CD8-double positive (DP). (c) CD8-single positive (SP). (d) CD4SP. Mice were sacrificed 7 days after the third irradiation and from 1 day to 3 weeks after the fourth irradiation. Six to fifteen mice were analyzed for each time point. Closed circles, irradiated infant mice; open circles, irradiated adult mice. Gray squares in each graph show the first or second regeneration period. *$P < 0.05$; †$P < 0.01$; ‡$P < 0.001$; §$P < 0.0001$, as compared with irradiated adult mice.

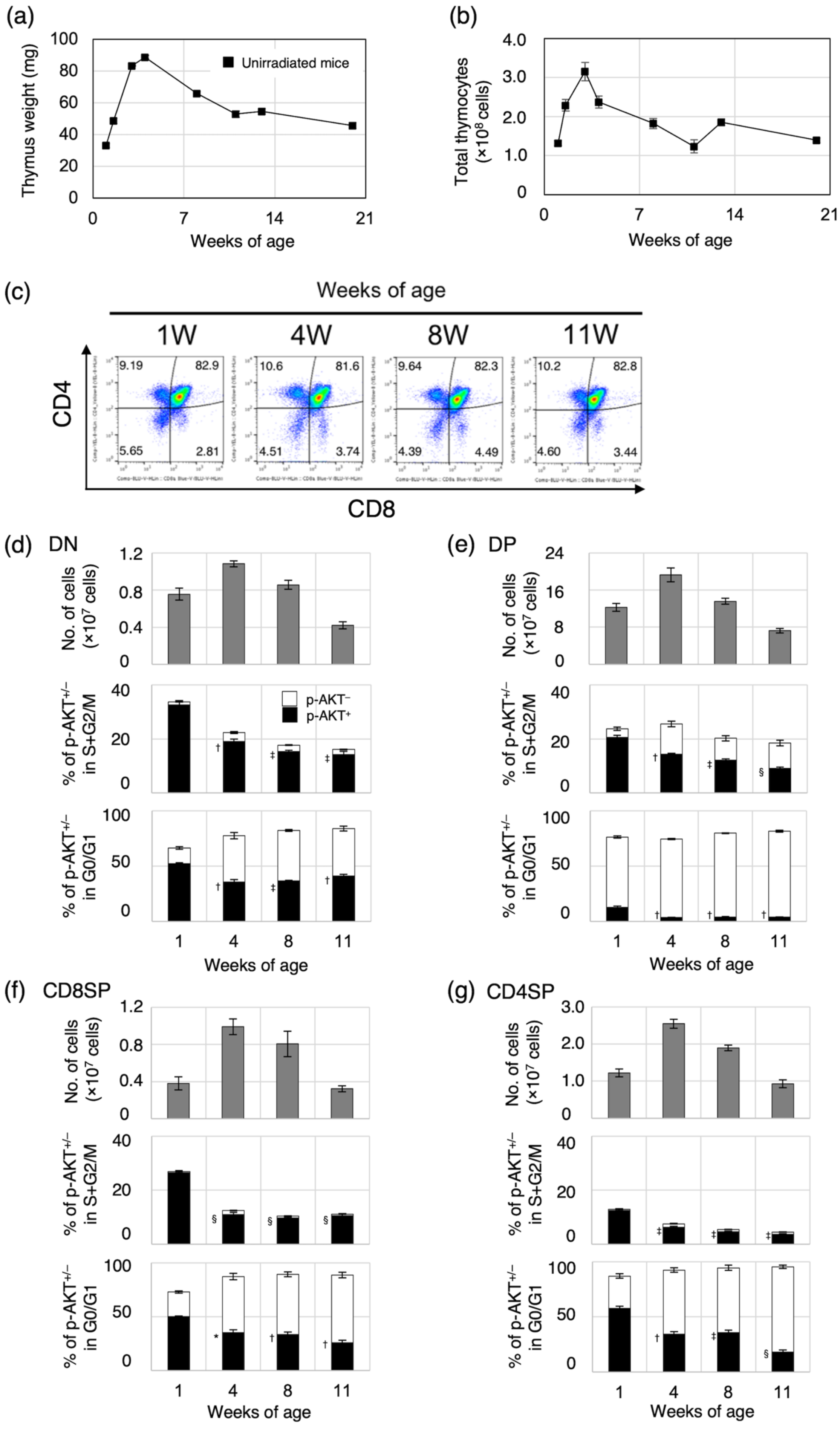

(a)
Thymus weight (mg)
Unirradiated mice
Weeks of age
(b)
Total thymocytes (×10⁸ cells)
Weeks of age
(c)
Weeks of age
1W
4W
8W
11W
CD4
CD8
9.19
82.9
5.65
2.81
10.6
81.6
4.51
3.74
9.64
82.3
4.39
4.49
10.2
82.8
4.60
3.44
(d) DN
(e) DP
No. of cells (×10⁷ cells)
% of p-AKT+/− in S+G2/M
% of p-AKT+/− in G0/G1
p-AKT−
p-AKT+
Weeks of age
(f) CD8SP
(g) CD4SP

Figure S6. Change in the number of cells and the proportion of phosphorylated-AKT-positive (p-AKT$^+$) thymocytes in non-irradiated mice at different ages. (a) Change in thymus weight. (b) Change in the number of thymocytes. (c) Representative profiles of CD4 and CD8 expression in thymocytes from mice at ages 1, 4, 8 and 11 weeks. (d) CD4 and CD8 double negative (DN), (e) CD4- and CD8 double positive (DP), (f) CD8-single positive (CD8SP), and (g) CD4SP. Top panels show the change in the number of cells in each differentiation stage. Middle panels show the proportion of p-AKT$^+$ or p-AKT$^−$ cells in the S+G2/M phase in each differentiation stage. Bottom panels show the proportion of p-AKT$^+$ or p-AKT$^−$ cells in the G0/G1 phase in each differentiation stage. Black bars, p-AKT$^+$ cells in the S+G2/M or G0/G1 phase; white bars, p-AKT$^−$ cells in the S+G2/M or G0/G1 phase. Three to eight mice were analyzed for each time point. $^*P < 0.05$; $^†P < 0.01$; $^‡P < 0.001$; $^§P < 0.0001$, as compared with the corresponding proportion of p-AKT$^+$ cells in mice at age 1 week.
